# Self-Compassion Components and Emotional Regulation Strategies as Predictors of Psychological Distress and Well-Being

**DOI:** 10.3390/bs15111576

**Published:** 2025-11-18

**Authors:** Sepideh Ranjouri, Denny Meyer, Glen William Bates

**Affiliations:** 1 Department of Psychological Sciences, Swinburne University of Technology, Melbourne, VIC 3122, Australia; sranjouri@swin.edu.au; 2 Department of Biomedical Health and Exercise Sciences, Swinburne University of Technology, Melbourne, VIC 3122, Australia

**Keywords:** self-compassion, emotion regulation, psychological distress, psychological well-being

## Abstract

Self-compassion is a positive self-related construct important in reducing symptoms of psychological distress and enhancing well-being. Self-compassion can be divided into compassionate self-responding (CSR), the ability to respond with self-kindness, a sense of common humanity, and mindfulness to one’s failures and negative experiences, and reduced uncompassionate self-responding (RUSR) the capacity to reduce self-judgment, isolation, and overidentification with emotional reactions. The current study was a preliminary investigation which examined the relationships of CSR and RUSR with psychological distress and well-being and explored the possible mediating effects on that relationship of emotional regulation via cognitive reappraisal and expressive suppression. A sample of 201 adults aged 18 to 73 from an Australian university completed an online survey. Structural equation modelling showed that compared to CSR, RUSR was a stronger predictor of psychological distress and eudaimonic well-being and a weaker predictor of hedonic well-being. Moreover, while emotion regulation strategies were found to mediate the relationships of CSR and RUSR with psychological distress and well-being, these relationships differed according to the outcome being predicted. The findings thus offer meaningful theoretical and treatment implications that provide direction for future research.

## 1. Introduction

Self-compassion is a self-related personality construct originally derived from Buddhist teachings that is defined as adopting a warm and non-judgmental mindset towards oneself in times of adversity and personal failure (Neff, 2003a; Gilbert, 2005). This mindset is linked to a capacity to recognize suffering and distress in oneself and in others combined with a deep commitment to alleviating that distress and enhance well-being (Gilbert et al., 2011). Over the last 20 years, psychological research on self-compassion has escalated and its importance in the determination of mental health and well-being is well established (Neff, 2023). A meta-analysis by MacBeth and Gumley (2012) reported a large aggregate effect size for associations between self-compassion and reduced psychological distress in terms of symptoms of anxiety, depression, and stress. Similarly, a meta-analysis by Zessin et al. (2015) found strong evidence that self-compassion is also associated with higher levels of psychological, cognitive, and affective well-being (Zessin et al., 2015).

Beyond the overall associations of self-compassion with psychological distress and well-being, however, the mechanisms whereby self-compassion reduces psychological distress and improves well-being remain under-researched (Finlay-Jones, 2017). In this study we explored the possible mediation role of emotion regulation strategies in the relationships between self-compassion and psychological distress and well-being. Emotion regulation can be generally defined as any conscious or unconscious action taken to change the valence and intensity of one’s current emotional experience (Gross, 2015). In essence, therefore, self-compassion constitutes a general form of problem solving as it generates motivation towards reducing negative emotions and increasing positive emotions (Berking & Whitley, 2014; Gilbert et al., 2011). Despite the theoretical link to emotion regulation, few studies have considered the relationship between self-compassion and emotion regulation strategies in relation to psychological distress and well-being. The aim of this preliminary study was to test the utility of predictive models of psychological distress and well-being which combined self-compassion with emotion regulation strategies as mediators.

### 1.1. Self-Compassion Components, Psychological Distress and Well-Being

The most prominent psychological model of self-compassion that is related to psychological distress and well-being was developed by Neff (2003a). Neff’s model comprises three interrelated components each with two opposite dimensions: positive and negative attitudes towards self. The components are: (1) self-kindness versus self-judgment, which refers to responding to oneself with kindness instead of criticizing oneself harshly in times of adversity; (2) common humanity versus isolation, referring to the capacity to view negative experiences as part of the unavoidable common nature of human beings instead of an individual’s unique misfortune; and (3) mindfulness versus overidentification, defined as accepting and trying to keep a balance between both negative and positive emotions and thoughts instead of exaggerating negative emotions.

Neff et al. (2018) distinguished compassionate self-responding—the ability to respond to adversity by utilizing positive components of self-compassion (self-kindness, common humanity, and mindfulness) from reduced uncompassionate self-responding, the capacity to reduce the influence of the negative components (self-judgment, isolation, and overidentification). In Neff’s measure of self-compassion (Neff, 2003b) the items for compassionate responding towards the self are worded positively (e.g., I’m tolerant of my own flaws and inadequacies) whereas the items related to reducing uncompassionate self-responding are worded negatively. These negatively worded items (e.g., When times are really difficult, I tend to be tough on myself) are reverse scored so that they combine with the compassionate self-responding score to provide an overall score.

Different strengths of relationship are evident between the two components of self-compassion with psychological distress, and well-being (Neff et al., 2018; Chio et al., 2021). In a meta-analysis involving 168 studies, Chio et al. (2021) found that correlations of the reduced uncompassionate self-responding component of self-compassion were stronger with psychological distress than were their compassionate self-responding counterparts. In contrast, the compassionate self-responding component had stronger correlations with well-being than the reduced uncompassionate self-responding components. This applied equally for separate measures of well-being derived from the two prominent definitions of well-being: subjective well-being and psychological well-being. Subjective well-being aligns with hedonic well-being and focuses on short-term pleasure-based happiness and general life satisfaction (Diener, 1984). Conversely, psychological well-being relates to eudaimonic well-being, enduring happiness derived from a sense of meaning and personal growth (Ryff, 1989).

Broadly consistent with Chio et al.’s findings, Neff et al. (2018) also found that reduced uncompassionate self-responding had a significantly stronger negative association than compassionate self-responding with symptoms including depression, anxiety, stress, and negative affect. However, in their study, compassionate self-responding and reduced uncompassionate self-responding showed similar positive and strong associations with positive psychological health outcomes including life satisfaction and positive affect.

The stronger relationship between reduced uncompassionate self-responding and psychopathology led Muris and Petrocchi (2017) to argue that the negatively worded components of the reduced uncompassionate self-responding (i.e., self-judgment, isolation, and overidentification with emotions) differ distinctly from compassionate self-responding and represent an attitude of ‘self-coldness’ unrelated to the positively worded items of compassionate self-responding (i.e., self-kindness, common humanity, and mindfulness). They concluded that the two components should not be combined into one construct. Further, Muris and Otgaar (2020) argued that combining the compassionate and reduced uncompassionate self-responding scales into a total scale measure of self-compassion falsely conflates the relationship of self-compassion with psychological symptoms and well-being. In response, Neff et al. (2018) argued that self-compassion is composed of positive responses to self (e.g., self-kindness) combined with an acceptance of negative aspects of the self which allows the person to refrain from uncompassionate self-responding (e.g., negative self-judgment). Neff (2023), therefore, sees the total self-compassion score as a holistic measure of self-compassion.

Recent psychometric evidence suggests that self-compassion can be regarded as a global single factor or as two factors without changing the definition of defining reduced uncompassionate self-responding to self-coldness. This retains the acceptance of negative aspects of the self within the theoretical basis of the test. Using a Bayes model analysis, Marsh et al. (2023) found that, although a two global factor model performed slightly better than a one-global factor model, the differences were not substantial. Neff (2020) and Marsh et al. (2023) concurred that the appropriate use of the SCS and its structural configuration should be determined by the aims of the researcher. In the present study, we examined the relationships for compassionate self-responding and reduced uncompassionate self-responding separately to investigate possible differences between the two forms of self-responding.

### 1.2. Emotion Regulation as a Mediator of the Relationship of Self-Compassion with Psychological Distress and Well-Being

Although limited, there is some evidence that difficulties in regulating emotions may mediate the effects of self-compassion on symptoms of psychological distress. A systematic review by Inwood and Ferrari (2018) identified five studies that had investigated this relationship. These studies used the total score for self-compassion and reported significant partial mediation of self-compassion by difficulties in regulating negative emotions for samples of participants with symptoms of depression, stress and post-traumatic stress. More recently, Bates et al. (2021) utilized Gross’s model (Gross, 2015; Gross & John, 2003) of emotion regulation to show that the specific regulation strategies of cognitive reappraisal and expressive suppression mediate self-compassion’s effects on social anxiety. Cognitive reappraisal is an adaptive cognitive change strategy referring to changing the interpretation of the situation from negative to neutral or positive (Diedrich et al., 2016). In contrast, expressive suppression is a maladaptive form of response modulation defined as the attempt to restrain or hide one’s ongoing emotion-expressive behavior rather than to show the felt emotion to others (Gross, 2015; Vally & Ahmed, 2020).

Bates et al. (2021) found that the compassionate self-responding component of self-compassion predicted a reduction in social anxiety but only through increased cognitive reappraisal. In contrast, the reduced uncompassionate self-responding component predicted lower social anxiety both directly and indirectly through reduced expressive suppression and increased cognitive reappraisal. Thus, for social anxiety, the mechanisms whereby the components of self-compassion, and its components reduce social anxiety, and the role of specific emotion regulation strategies was complex. However, to date, no study has investigated emotion regulation strategies as mediators of the relationships of the components of self-compassion with general psychological distress or with hedonic and eudaimonic well-being.

### 1.3. The Current Study

**Figure 1:**
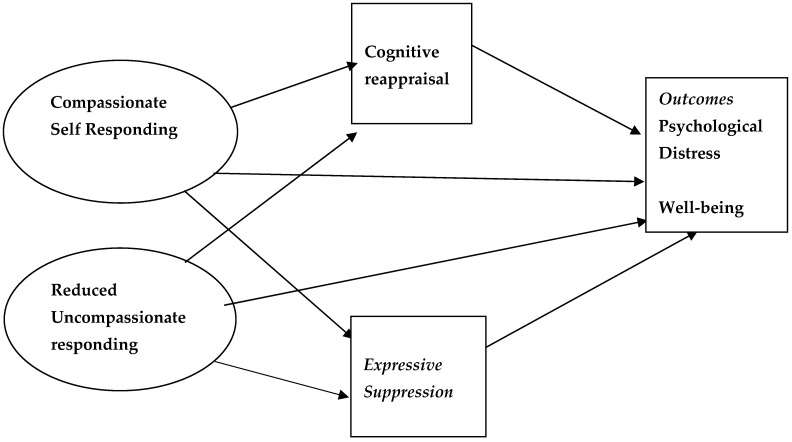
General model of prediction of psychological distress and well-being.

The present study was a preliminary investigation in which we used structural equation modelling to investigate possible differences between the two self-compassion components (reduced uncompassionate self-responding and compassionate self-responding) in predicting psychological distress and hedonic and eudaimonic well-being. Based on theoretical links among the concepts we also explored the role of the emotion regulation strategies of cognitive reappraisal and expressive suppression as mediators in the relationships of the two self-compassion components with psychological distress and well-being. A general model of the proposed relationships among the variables is presented in Figure 1.

Building on previous research, it was hypothesized that:

**Hypothesis** **1.** *Reduced uncompassionate self-responding would be a stronger negative predictor of psychological distress than compassionate self-responding.*

**Hypothesis** **2.** *Compassionate self-responding would be a stronger positive predictor of both forms of well-being than reduced uncompassionate self-responding.*

**Hypothesis** **3.** *Cognitive reappraisal would positively mediate the relationship of compassionate self-responding and reduced uncompassionate self-responding with both forms of well-being and negatively mediate the relationship of compassionate self-responding and reduced uncompassionate self-responding with psychological distress.*

**Hypothesis** **4.** *Expressive suppression would negatively mediate the relationship of compassionate self-responding and reduced uncompassionate self-responding with both forms of well-being and positively mediate the relationship of compassionate self-responding and reduced uncompassionate self-responding with psychological distress.*

## 2. Method

### 2.1. Participants and Procedure

Secondary data were acquired from the Swinburne Student Self-Assessment Survey. The survey was accessible to all Higher and Further Education students at Swinburne University of Technology during 2019 and 2020. All enrolled students received an email describing the survey with a link to the survey containing several well-being, study style, and employability measures. Information about consent to participate and the use of deidentified data in future studies was provided. Participating was voluntary, and participants could withdraw at any time up until they submitted their survey. They were also informed about Swinburne student support services and received personalized feedback and downloadable self-help materials. Students took approximately 60 min to complete the survey. The final convenience sample comprised 201 adult students in Higher and Further Education (118 women, 81 men, and two other) aged 18 to 73 years (*M* = 26.13, *SD* = 9.50). The students came from a range of faculties across the university (43.8% Health Arts and Design; 30% Science and Engineering; 14.9% Business and Law; and 10.9% Vocational Education). In terms of employment, 45% of the sample were full-time students; 21% were studying while employed casually or part-time; and 11% were studying while working full-time. Their living arrangements were reported as living at home with parents (41%); living independently (55%); or ‘other arrangements’ (11%).

### 2.2. Measures

*The self-compassion scale* (SCS, Neff, 2003b) is a 26-item questionnaire comprised of six subscales measuring the three main bipolar components of self-compassion. Respondents rate how often they behave in the stated manner on a 5-point Likert scale from 1 (Almost never) to 5 (Almost always). In scoring, the negatively worded items are reverse coded. The total score for each subscale is the mean of its items. The overall total score is the sum of six total scores of each subscale. The sum of the total scores of self-kindness, common humanity, and mindfulness forms the Compassionate Self-responding score. The sum of reverse-coded scores of self-judgment, isolation, and overidentification is the reduced uncompassionate self-responding score. Neff (2003b) reported high internal consistency (*α* = 0.93) for the SCS and high test–retest reliability. The SCS also shows good construct, content, and convergent validity (Neff, 2016). In the present study, Cronbach’s alpha showed excellent internal consistency for the total score (0.93) and for the two subscales (CSR = 0.93; RUSR = 0.92).

*Emotion Regulation Scale* (ERS; Gross & John, 2003) is a 10-item questionnaire measuring the strategies of cognitive reappraisal and expressive suppression Respondents rate how they manage their emotions by identifying their level of agreement to statements on a 7-point Likert scale from 1 (*Strongly agree*) to 7 (*Strongly disagree*). The ERS shows good internal consistency for both subscales across four samples (ES averaged *α* = 0.73 and CR averaged *α* = 0.79), good test–retest reliability (0.69) over three months, and good validity (Gross & John, 2003). In the present study, Cronbach’s alpha showed excellent internal consistency for cognitive reappraisal (0.90) and moderate reliability for expressive suppression (0.73).

### 2.3. Measures of Psychological Distress

*Depression Anxiety and Stress Scale* 21 item version (DASS-21; Lovibond & Lovibond, 1995) is a short form of DASS-42, a questionnaire consisting of three subscales measuring depression, anxiety, and stress. Each subscale comprises seven items. Respondents indicate how much the item has applied to them over the past week on a 4-point Likert scale from 0 (*Not at all*) to 3 (*Very much, or most of the time*). All items are summed to compute a total score. DASS-21 has demonstrated excellent internal consistencies (*α* = 0.93) and adequate construct validity (Henry & Crawford, 2005). In the present study, the Cronbach alpha for the total DASS-21 was excellent (0.95).

*Negative Version of Positive and Negative Affect Schedule* (NA-PANAS; Watson et al., 1988) is a 10-item questionnaire measuring negative affect. Respondents rate their emotional experience over the past three months on a 5-point Likert scale from 1 (*very slightly or not at all*) to 5 (*very much*). Crawford and Henry (2004) reported good reliability for the negative affect version of PANAS (*α NA* = 0.85) and strong convergent validity. In the present study, internal consistency was excellent (alpha = 0.88).

### 2.4. Measures of Well-Being

*Positive Version of PANAS* (PA-PANAS; Watson et al., 1988) is a 10-item scale measuring positive affect, an index of subjective well-being. Respondents rate their positive emotional experiences over the past three months on a 5-point Likert scale from 1 (*very slightly or not at all*) to 5 (*very much*). The positive affect scale shows good reliability (*αPA* = 0.89) and strong convergent validity (Crawford & Henry, 2004). In the current study, internal consistency was excellent (alpha = 0.90).

*Satisfaction With Life Scale* (SWLS; Diener et al., 1985) is a five-item measure measuring SWB. Respondents rate how much they agree with statements on a 5-point scale from 1 (*Strongly disagree*) to 5 (*Strongly agree*). SWLS has shown good internal consistency (*α* range = 0.86 to 0.87; Bauer et al., 2008), good test–retest reliability (0.83) in two weeks (Gouveia et al., 2009), and convergent validity (Pavot & Diener, 2008). In the current study, internal consistency was excellent (alpha = 0.90).

*Psychological Well-Being Scale* (PWBS; Ryff, 1989; Ryff & Keyes, 1995): Eudaimonic well-being was measured by a 36-item version of the PWBS which consists of six subscales. Respondents rate the extent to which each statement describes them on a 6-point Likert scale from 1 (*Strongly Disagree*) to 6 (*Strongly Agree*). A total score is calculated by reverse scoring negatively worded items and summing all other items. The PWBS has demonstrated excellent internal consistency (*α* range = 0.94 to 0.95) and good construct validity (Bauer et al., 2008; Whitehead & Bates, 2016). In the current study, internal consistency was excellent (alpha = 0.92).

### 2.5. Statistical Analysis

To test the hypotheses, four structural equation models (SEMs) were conducted using MPlus version 6. Compassionate self-responding and reduced uncompassionate self-responding were created as latent variables by combining the three relevant subscales for each component and the measures of psychological distress and well-being were used as endogenous variables with cognitive reappraisal and expressive suppression included as mediators. The general predictive model is illustrated in Figure 1.

One SEM predicted scores on positive affect as a measure of hedonic well-being and scores on negative affect as a measure of psychological distress. A second SEM predicted total scores the DASS-21 as a composite measure of psychological distress. A third SEM predicted scores on the psychological well-being scale as an indicator of eudaimonic well-being. A fourth SEM predicted scores on the subjective well-being scale as another measure of hedonic well-being. In each SEM, acceptable model fit was determined according to the specifications of Hu and Bentler (1999). These were: Goodness-of-Fit Index (GFI), Comparative Fit Index (CFI) and Tucker–Lewis Index (TLI) ≥ 0.95, Root Mean Score Error Approximation (RMSEA) ≤ 0.06.

A power analysis using G*power Version 3.1.9.7 estimating 12 parameters for each of the latent constructs (CSR and RUSR) estimated a required sample size of 157 for single item estimates (DASS-21, PWB) and 175 for two outcome measures (PA and NA). In addition, we considered general rules of thumb estimates of sample size. Kline (2015) recommended a minimum of 20 participants per variable for reliable SEM predictions. Although other studies suggest 10:1 or 5:1 participant-to-variables ratios are sufficient, using the 20:1 ratio proposed by Kline (2015) allows for a more conservative approach. This provided estimates of 120 for the two-outcome model (PA and NA) and 100 for the single outcome models (DASS-21 and PWB).

## 3. Results

### 3.1. Data Screening

No multivariate and univariate outliers or missing values were identified. The normality assumption was violated for NA, DASS-21, and cognitive reappraisal. These variables were transformed, and analyses were run with and without transformation. Pearson correlations showed no differences in correlations between the transformed and untransformed NA, DASS-21, and cognitive reappraisal with other variables. Therefore, untransformed variables were used in correlation analyses. SEM analyses were also conducted with transformed and untransformed variables. No differences were found between transformed and untransformed NA and CR. However, transformed DASS-21 (SQRT-DASS-21) was a better fitting variable, so it was used in SEM analysis. The linear relationship assumption was checked for all variables using scatterplots and no non-linear relationships were found.

Given the wide age range of 18 to 73 and a mean age of 26.13, we repeated our SEM analyses with a sample ranging in age from 18–36 (mean age = 24.14). This reduced the sample size to 177, which was still over the number specified in our power analysis (175 for two outcomes of PA and NA) and 157 for the single outcome models (DASS-21 and PWB). As the results of all analyses duplicated those with the full sample, we have reported the full sample statistics here and have included the reduced sample analyses in Supplementary Document S1. We also checked on the correlation of age with all variables which were negligible (0.046 to 0.181), further suggesting our findings were not influenced by the age composition of the sample.

### 3.2. Correlations Among the Measures

Descriptive statistics, reliabilities, and correlations for all variables are displayed in Table 1. As shown in Table 1, reliability was high to excellent for all measures except for expressive suppression (moderate). The correlations among the variables showed significant, strong negative relationships between RUSR and the indicators of psychological distress (NA and DASS-21) that were stronger than the associations of CSR with the psychological distress indicators. In contrast, significant, moderate positive relationships were evident for CSR and the mental well-being indicators (PA, SWLS, PWBS), but their associations were little different to those with RUSR, except for PWBS, which showed a somewhat stronger association with RUSR (*r* = 0.61) compared to CSR (*r* = 0.47).

### 3.3. Prediction of Negative Affect and Positive Affect

**Figure 2:**
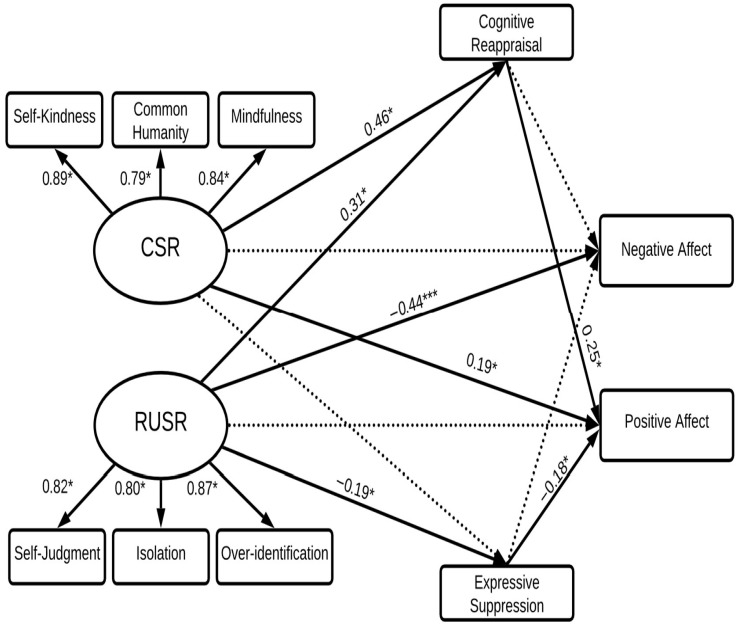
Structural equation model predicting negative and positive affect. *Note.* This structural equation model predicts negative and positive affect from CSR and RUSR, with mediating effects of cognitive reappraisal and expressive suppression. CSR = compassionate self-responding; RUSR = reduced uncompassionate self-responding. Dotted lines represent non-significant relations; bold lines represent significant relations.* *p* < 0.05; *** *p* < 0.001.

As low negative affect is taken as an indicator of hedonic well-being as well as being an indicator of psychological distress when high, negative and positive affect were explored as two related outcome variables in SEM to test hypotheses (see Figure 2). The significance of the relevant indirect pathways for positive affect and negative affect are presented in Table 2.

The overall model was a good fit to the data (*χ*^2^(24) = 1.58, GFI = 0.96, CFI = 0.99, TLI = 0.97, RMSEA = 0.05). CSR and RUSR directly predicted 39% of the variance in negative affect and positive affect indirectly through cognitive reappraisal and expressive suppression.

*Prediction of NA*. As expected (Hypothesis 1), RUSR was a stronger negative predictor of NA than CSR. The direct pathway between RUSR and NA (*β* = −0.44) was significant whereas the direct pathway between CSR and NA was non-significant.

Hypotheses 3 and 4 were tested by considering the mediating effect of cognitive reappraisal and expressive suppression on the relationships of CSR and RUSR with NA. Contrary to expectation, CR and ES did not mediate the relationships of CSR or RUSR with NA. Thus, NA was predicted solely by the direct effect of RUSR.

*Prediction of PA.* To test Hypothesis 2, direct pathways were compared between CSR, RUSR and PA as an indicator of hedonic well-being. As shown in Figure 1, the direct pathway between CSR and PA (*β* = 0.19) was significant. However, the direct pathway of RUSR and PA was non-significant. Thus, as hypothesized CSR was a stronger positive predictor of PA than RUSR.

Hypotheses 3 and 4 were tested by considering the mediating effects of cognitive reappraisal and expressive suppression on the relationships of CSR and RUSR with PA. As shown in Table 2, consistent with Hypothesis 3, the indirect pathway between CSR and PA via Cognitive Reappraisal (*β* = 0.11) was significant and so was the indirect pathway between RUSR and PA via cognitive reappraisal (*β* = 0.08). Hypothesis 4 received partial support. Whereas the indirect pathway between RUSR and PA through expressive suppression was significant (*β* = 0.07) the indirect pathway between CSR with PA through expressive suppression was non-significant. Therefore, compassionate self-responding predicted PA directly and indirectly via cognitive reappraisal but not expressive suppression. In contrast, reduced uncompassionate behavior did not directly predict PA but did so indirectly through its full mediation by cognitive reappraisal and expressive suppression.

### 3.4. Prediction of Psychological Distress

**Figure 3:**
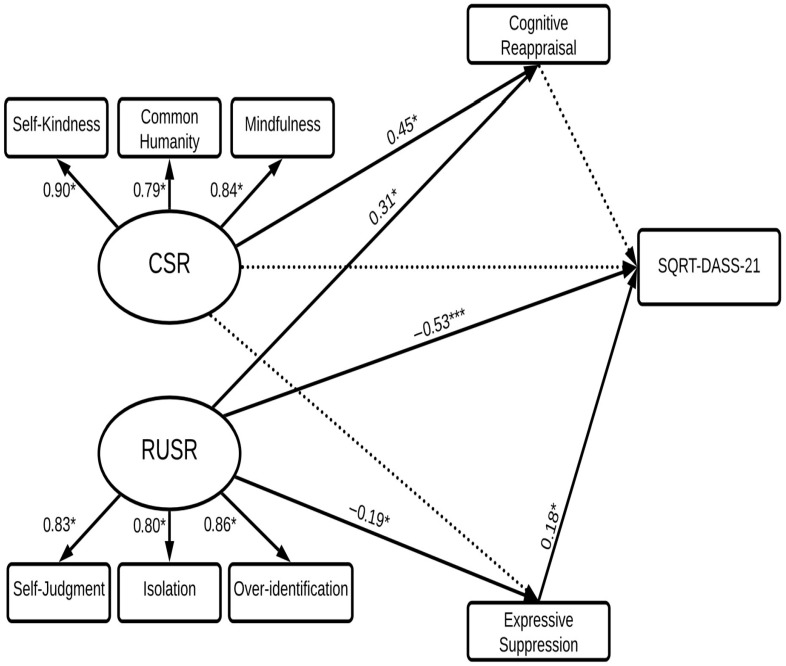
Structural equation model predicting DASS-21. *Note.* This structural equation model predicts DASS-21 from CSR and RUSR, with mediating effects of cognitive reappraisal and expressive suppression. CSR = compassionate self-responding; RUSR = reduced uncompassionate self-responding; SQRT-DASS-21 = Square Root Depression, Anxiety, Stress Scale. Dotted lines represent non-significant relations; bold lines represent significant relations.* *p* < 0.05; *** *p* < 0.001.

A second SEM was used for DASS-21 as another measure of psychological distress (see Figure 3). Results for the indirect pathways are presented in Table 2.

The model was a good fit to the data (*χ*^2^(24) = 1.90, GFI = 0.96, CFI = 0.98, TLI = 0.97, RMSEA = 0.07). CSR and RUSR directly predicted 50% of the variance in DASS-21 indirectly through cognitive reappraisal and expressive suppression. As predicted (Hypothesis1), RUSR was a stronger negative predictor of DASS-21 than CSR. The direct pathway between RUSR and SQRT-DASS-21 (*β* = −0.53) was significant. However, the direct pathway between CSR and SQRT-DASS-21 was non-significant.

To assess Hypotheses 3 and 4 for DASS-21 the indirect pathways between CSR and RUSR in predicting SQRT-DASS-21 were examined. Contrary to prediction (Hypothesis 3) cognitive reappraisal did not mediate the effects of either CSR or RUSR. However, in partial support of Hypothesis 4, expressive suppression mediated the influence of RUSR on SQRT-DASS-21 (*β* = −0.07). However, contrary to expectation, the indirect influence of CSR via expressive suppression was non-significant.

### 3.5. Prediction of Psychological Well-Being (Eudaimonic Well-Being)

**Figure 4:**
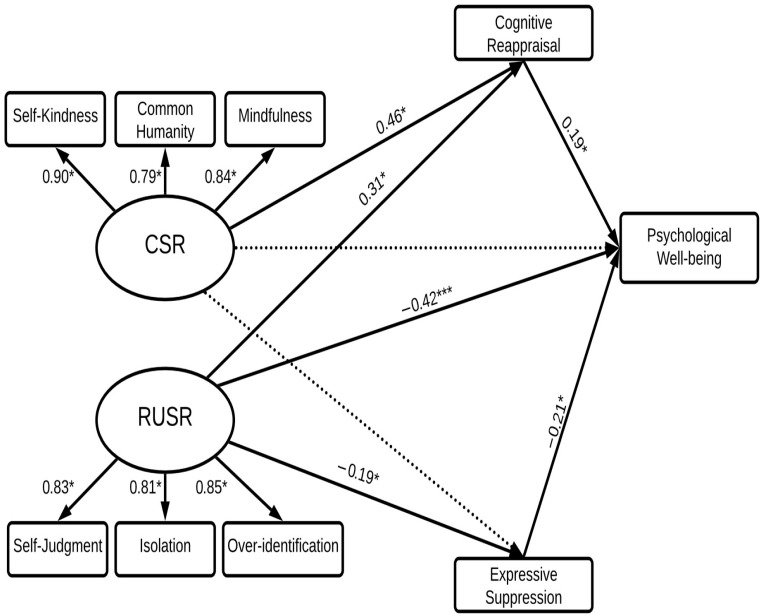
Structural equation model predicting psychological well-being. *Note.* This structural equation model predicts psychological well-being from CSR and RUSR, with mediating effects of cognitive reappraisal and expressive suppression. CSR = compassionate self-responding; RUSR = reduced uncompassionate self-responding. Dotted lines represent non-significant relations; bold lines represent significant relations.* *p* < 0.05; *** *p* < 0.001.

To investigate the hypotheses for PWB as another measure of eudaimonic well-being, a third SEM was conducted for PWBS (see Figure 4). Results for the indirect prediction of PWB are in Table 2.

The model was a good fit to the data (*χ*^2^(24) = 2.05, GFI = 0.96, CFI = 0.98, TLI = 0.96, RMSEA = 0.07). CSR and RUSR directly predicted 51% of the variance in PWBS indirectly through CR and ES. Contrary to expectation (Hypothesis 2), RUSR was a stronger positive predictor of PWB than CSR. While the direct pathway between RUSR and PWBS (*β* = 0.42) was significant, the direct pathway between CSR and PWBS was non-significant.

To examine Hypotheses 3 and 4, indirect pathways from CSR, RUSR to PWBS through cognitive reappraisal and expressive suppression were compared. Consistent with Hypothesis 3, cognitive reappraisal was a significant positive mediator of the relationship between CSR and PWBS (*β* = 0.09) and between RUSR and PWBS (*β* = 0.06). However, Hypothesis 4 received only partial support. Consistent with expectation, the indirect pathway between RUSR and PWBS via expressive suppression (*β* = 0.04) was significant. As shown in Figure 3, there were negative effects between RUSR and expressive suppression (*β* = −0.19) and between ES and PWBS (*β* = −0.21). However, in SEM the indirect effect of one variable via the influence of another is represented by the multiplication of the *β*’s for the variables involved (Baron & Kenny, 1986). Therefore, as shown in Table 2, the positive direction of ES in the indirect pathway given by MPlus between RUSR and PWBS results from the multiplication of two negative numbers and appears as positive. Taken together, therefore, expressive suppression acted as a negative mediator in the association of RUSR with PWBS. In contrast to RUSR, for CSR, the results were contrary to Hypothesis 4 as expressive suppression was not a significant mediator.

### 3.6. Results of SEM for Subjective Well-Being

A SEM for SWLS as another indicator of hedonic well-being not a good fit to the data (*χ*^2^(24) = 3.20, GFI = 0.94, CFI = 0.95, TLI = 0.92, RMSEA = 0.10) and SWLS was highly correlated with other variables. For this reason, we did not consider the findings for this model in the results.

## 4. Discussion

This study was a preliminary investigation that examined an integrative model of the relationships of compassionate self-responding and reduced uncompassionate self-responding with psychological distress and with hedonic and eudaimonic well-being and the possible mediating effects of cognitive reappraisal and expressive suppression on these associations. Overall, the findings provided meaningful insights into the relationships among the variables which were broadly consistent with previous research on specific aspects of the model or offered new insights into the relationships. As expected (Hypothesis 1), reduced uncompassionate self-responding was a stronger predictor of decreased psychological distress than compassionate self-responding. Also consistent with prediction (Hypothesis 2), compassionate self-responding was a stronger predictor of higher levels of hedonic well-being (positive affect) than reduced uncompassionate self-responding. However, contrary to expectation, compassionate self-responding did not directly predict psychological well-being (eudaimonic well-being) whereas reduced uncompassionate self-responding significantly predicted higher psychological well-being. Also, against prediction (Hypothesis 3), neither cognitive reappraisal nor expressive suppression mediated the relationships of the two forms of self-compassion with negative affect and only expressive suppression mediated the relationship with psychological distress (SQRT-DASS-21). However, consistent with Hypothesis 4, higher cognitive reappraisal and lower expressive suppression mediated the associations of compassionate self-responding and reduced uncompassionate self-responding with higher positive affect and higher psychological well-being. The implications of the findings are discussed separately for psychological distress and mental well-being.

### 4.1. Psychological Distress Findings

That reduced uncompassionate self-responding directly predicted lower general psychological distress and negative affect but compassionate self-responding reflects a more pronounced difference between the two components of self-compassion than in earlier work. Neff et al. (2018) reported that although the relationship between reduced uncompassionate self-responding and symptoms of psychological distress was stronger both components predicted symptoms. Thus, our findings suggest that the ability to reduce critical self-judgment, to resist a sense that others are better off than oneself (Isolation) and not to become immersed in negative feelings (overidentification) are the primary mechanisms whereby self-compassion can reduce distress. This is underscored by our finding that lower reduced uncompassionate self-responding also predicted less emotional suppression which provided an additional indirect pathway to lower psychological distress. Thus, an ability to express emotional experiences combined with reduced uncompassionate self-responding appears to be a primary locus of effective management of psychological distress. On the other hand, compassionate self-responding did not predict expressive suppression and showed no indirect influence on psychological distress through ethe emotion regulation strategies.

That cognitive reappraisal did not mediate the relationships of compassionate self-responding or reduced uncompassionate self-responding with psychological distress despite its strong negative correlation is inconsistent with previous research on social anxiety (Bates et al., 2021; Dryman & Heimberg, 2018). Bates et al. (2021) is the most comparable study to our study as they used the same emotion regulation strategies and components of self-compassion. As in the present study, Bates et al. found no direct effect for compassionate self-responding on social anxiety but reported a significant direct effect for reduced uncompassionate self-responding. However, they found partial mediation by cognitive reappraisal as well as expressive suppression for two separate measures of social anxiety. Increased compassionate self-responding predicted higher levels of cognitive appraisal, which in turn predicted lower social anxiety. These different findings may be due to the nature of social anxiety and its difference from general psychological distress. The crucial factor in social anxiety is identified as a fear of negative evaluation of the self by others (American Psychiatric Association, 2022). This accentuates attitudes towards the self in determining emotional responses to difficult social situations (e.g., critical self-judgment, or self-kindness). In contrast, our measure of psychological distress (DASS-21) focuses on specific signs of distress (e.g., I felt downhearted and blue; I was aware of dryness in my mouth). This suggests that the relationship of self-compassion and emotion regulation via cognitive reappraisal may differ across mental health conditions and populations. This possibility invites further research comparing specific forms of psychological distress (e.g., depression, generalized anxiety or symptoms of PTSD).

### 4.2. Findings for Hedonic and Eudaimonic Well-Being

The findings for well-being were more complex than those for psychological distress. compassionate self-responding predicted higher levels of hedonic well-being as measured by positive affect, whereas reduced uncompassionate self-responding did not. However, for the other aspect of hedonic well-being, lower levels of negative affect, the reverse applied. Compassionate self-responding did not predict lower negative affect, whereas reduced uncompassionate self-responding directly predicted lower negative affect. This pattern of findings is consistent with the results of a meta-analysis by Chio et al. (2021) which found compassionate self-responding had a stronger relationship with positive hedonic well-being than reduced uncompassionate self-responding. Thus, as per the broaden-and-build theory (Fredrickson, 2001), compassionate self-responding may raise positive emotions that expand one’s perspective and assist the individual to build a wide range of personal resources leading to positive state of mental well-being.

Our results for hedonic well-being also showed a unique pattern in how the emotion regulation strategies influenced the relationship between the self-compassion components and hedonic well-being. Both reduced expressive suppression and increased cognitive reappraisal enhanced the influence of reduced uncompassionate self-responding on increased experiences of positive affect. In addition, increased cognitive reappraisal increased the influence of compassionate self-responding on positive affect. Interestingly, neither of the mediation pathways were significant for decreasing negative affect. This suggests that specific strategies of emotion regulation are most effective in raising the frequency of positive affective states. This aligns with Neff’s model (Neff, 2003a), which proposes that self-compassion assists individuals to prevent themselves from experiencing suffering in the first place by adopting proactive behaviors (such as cognitive appraisal and reduced expressive suppression) aimed at experiencing positive affect. As this did not apply to decreasing negative affect it may be that these effects may be confined to positive affective experiences.

Interestingly the results for psychological well-being (eudaimonic well-being) differed somewhat from those for hedonic well-being. The pattern of results for reduced uncompassionate self-responding showed a significant direct effect and significant indirect effects through both mediators (cognitive reappraisal and expressive suppression). In contrast, compassionate self-responding’s influence on eudaimonic well-being was fully determined by its relationship with cognitive reappraisal. Thus, compassionate responses to the self, such as self-kindness or keeping a balanced mind, appear to require an established ability to reappraise and alter negative emotions to more positive emotions to be effective. This, therefore, further emphasizes the importance of reducing uncompassionate behaviors in creating more of a sense of purpose and meaningful relationships as well as in increasing experiences of positive emotions. It also suggests that decreasing expressive suppression of emotions and increasing cognitive appraisal to alter emotions towards positive rather than negative emotions are important in this process. As these possible relationships among the variables have not previously been explored, they invite further research.

### 4.3. Clinical Implications, Methodological Considerations, and Directions for Future Research

Our results have implications for the development of self-compassion interventions. For individuals experiencing high levels of psychological distress, interventions may be enhanced by focusing more upon developing the client’s capacity to reduce uncompassionate self-responding and expressive suppression of emotions than on increasing compassionate self-responding. However, in public mental health promotion strategies targeting the general populace, increasing compassionate self-responding and the ability to engage in cognitive appraisal is also likely to increase experiences of positive affect and increase psychological (eudaimonic) well-being.

A strength of this study was the integration of two theoretical models (self-compassion, and emotional regulation) into a framework to predict psychological distress and mental well-being. In addition, the study employed well established self-report measures with high reliability and validity. The sample also had a good balance of students across different university faculties. However, several limitations need to be considered. First, the sample was a non-clinical and well-educated convenience sample of university students. Moreover, the participants in the sample were concentrated in younger age groups and 59% of the sample was female. This means the results cannot be readily generalized to community and clinical populations. Also, although the sample size in the SEM models met the base levels required for 90% confidence, a larger sample would provide greater confidence in the estimates generated. As this was a preliminary study of the proposed relationships among the measures it is encouraging that we obtained excellent fit for our models using conservative fit indices and the results were theoretically meaningful. However, replication of the findings is now needed with a larger sample and with a broader general community and clinical samples. Related to this is the limitation that this study employed a cross-sectional design which precludes causal interpretations of the relationships among the variables. Longitudinal studies are also needed, therefore, to confirm the directionality of the relationships we have found.

Another limitation of this study was that measurement of the variables was restricted to self-report measures. Although the measures we used are well validated and extensively researched, they remain subject to biases and limitations such as social desirability. Behavioral measures and experimental research in various contexts would help to establish the role of self-compassion components in facilitating emotions (Inwood & Ferrari, 2018; McBride et al., 2022). For example, in addition to self-reports, structural clinical interviews, and behavioral indices of emotion dysregulation in ecologically valid contexts would help verify and extend our understanding of the relationships among the variables. Moreover, our measurement of emotional regulation strategies was confined to cognitive reappraisal and expressive suppression. Although these are important strategies, there are many other aspects of emotion regulation that are relevant to psychological distress and well-being. Other forms of emotion regulation that have been shown to influence self-compassion, anxiety and depression include acceptance of emotions, problem solving, avoidance and rumination (Cutajar & Bates, 2025). In addition, these strategies are confined to the intrapersonal aspects of emotional regulation (efforts made by people themselves). Another conceptualization of emotion regulation includes strategies which regulate emotions extrinsically by involving other people (Hofmann, 2014). Hofmann et al. (2016) identified interpersonal strategies such as perspective taking and social modelling (i.e., seeking others’ evaluations of the situation and hearing how they have handled similar situations) and soothing (receiving emotional support from others) as important sources of emotion regulation that augment the person’s own internal strategies. Future research including interpersonal and interpersonal components of emotion regulation is likely to provide a useful extension of our findings.

In conclusion, the present study was an initial investigation and the first to explore the mediation role of emotion regulation strategies in the relationships between self-compassion components with psychological distress and well-being. We found differences between compassionate self-responding and reduced uncompassionate self-responding in the prediction of psychological distress and with different forms of well-being. These confirm the greater involvement of reduced uncompassionate self-responding in predicting psychological distress and eudaimonic well-being. We also found that the emotion regulation strategies of cognitive reappraisal and expressive suppression acted as mediators of the self-compassion components although these mediation relationships varied according to the outcome being predicted. Our findings are preliminary and invite replication in larger longitudinal samples to establish the causality of the observed relationships.

## Figures and Tables

**Table 1:** Descriptive statistics, Cronbach’s alpha reliability coefficient, and correlations for all study variables.

Variable	*M*	*SD*	*α*	1	2	3	4	5	6	7	8	9	10
1. NA-PANAS	25.26	8.50	0.88	-									
2. DASS-21	19.46	14.13	0.95	0.70 **	-								
3. PA-PANAS	34.67	8.18	0.90	−0.32 **	−0.47 **	-							
4. PWBS	153.31	26.54	0.92	−0.60 **	−0.71 **	0.69 **	-						
5. SWLS	15.26	5.19	0.90	−0.49 **	−0.49 **	0.42 **	0.63 **	-					
6. CR-ERS	28.00	7.50	0.90	−0.43 **	−0.46 **	0.45 **	0.51 **	0.30 **	-				
7. ES-ERS	15.60	5.18	0.73	0.19 **	0.28 **	−0.22 **	−0.30 **	−0.21 **	0.04	-			
8. SCS	17.96	4.37	0.93	−0.57 **	−0.60 **	0.48 **	0.63 **	0.48 **	0.64 **	−0.18 **	-		
9. CSR-SCS	9.20	2.44	0.92	−0.41 **	−0.40 **	0.43 **	0.47 **	0.36 **	0.58 **	−0.10	0.85 **	-	
10. RUSR-SCS	8.79	2.65	0.92	−0.56 **	−0.63 **	0.40 **	0.61 **	0.45 **	0.52 **	−0.20 **	0.87 **	0.87 **	-

*Note*. *N* = 201. 1 = NA-PANAS = Negative Affect-Positive and Negative Affect Scale; 2 = DASS-21 = Depression, Anxiety, Stress Scale-21; 3 = PA-PANAS = Positive Affect-Positive and Negative Affect Scale; 4 = PWBS = Psychological Well-Being Scale; 5 = SWLS = Satisfaction With Life Scale; 6 = CR-ERS = Cognitive Reappraisal-Emotion Regulation Scale; 7 = ES-ERS = Expressive Suppression-Emotion Regulation Scale; 8 = SCS = Self-Compassion Scale; 9 = CSR-SCS = Compassionate Self-Responding- Self-Compassion Scale; 10 = RUSR-SCS = Reduced Uncompassionate Self-Responding-Self-Compassion Scale; ** *p* < 0.01.

**Table 2:** Indirect pathway analyses for three sets of modelled effects.

Pathway Tested	Indirect Effect
Negative Affect with CSR	
CSR > Cognitive Reappraisal > Negative Affect	−0.04
CSR > Expressive Suppression > Negative Affect	0.00
Negative Affect with RUSR	
RUSR > Cognitive Reappraisal > Negative Affect	−0.03
RUSR > Expressive Suppression > Negative Affect	−0.02
Positive Affect with CSR	
CSR > Cognitive Reappraisal > Positive Affect	0.11 **
CSR > Expressive Suppression > Positive Affect	0.00
Positive Affect with RUSR	
RUSR > Cognitive Reappraisal > Positive Affect	0.08 *
RUSR > Expressive Suppression > Positive Affect	0.07 *
Psychological distress with CSR	
CSR > Cognitive Reappraisal > Psychological distress	−0.06
CSR > Expressive Suppression > Psychological Distress	0.00
Psychological Distress with RUSR	
RUSR > Cognitive Reappraisal > Psychological Distress	−0.04
RUSR > Expressive Suppression > Psychological Distress	−0.07 *
Psychological well-being with CSR	
CSR > Cognitive Reappraisal > Psychological Well-Being	0.09 *
CSR > Expressive Suppression > Psychological Well-Being	0.00
Psychological Well-Being with RUSR	
RUSR > Cognitive Reappraisal > Psychological Well-Being	0.06 *
RUSR > Expressive Suppression > Psychological Well-Being	0.04 *

*Note*. CSR = compassionate self-responding; RUSR = reduced uncompassionate self-responding;. Psychological distress is measured by SQRT-DASS-21. * *p* < 0.05; ** *p* < 0.01.

## Data Availability

The raw data supporting the conclusions of this article will be made available by the authors on request.

## Supplementary Materials

The following supporting information can be downloaded at: https://www.mdpi.com/article/10.3390/bs15111576/s1, Supplementary Document S1: Models for people aged 18–35. Figure S1: Structural Model with Standardised Weights and Significant Paths Bolded. Figure S2: Structural Model with Standardised Weights and Significant Paths Bolded. Figure S3: Structural Model with Standardised Weights and Significant Paths Bolded. Figure S4: Structural Model with Standardised Weights and Significant Paths Bolded. Table S1: Standardised Effect Sizes. Table S2: Standardised Effect Sizes. Table S3: Standardised Effect Sizes. Table S4: Standardised Effect Sizes.
