# Supplementary material for: Self-Compassion Components and Emotional Regulation Strategies as Predictors of Psychological Distress and Well-Being
Source: Behav Sci (Basel). 2025 Nov 18;15(11):1576. doi: 10.3390/bs15111576 (PMC12649398; doi:10.3390/bs15111576)
Supplement: Supplementary file 1 [file behavsci-15-01576-s001.zip › behavsci-3883859-supplementary.pdf]

Internal

Supplementary Materials

Models for people aged 18-35

PANAS Model describes the data reasonably well

(Normed Chi-Square = 1.847579, GFI=.953, TLI=.955, CFI=.976, RMSEA=.070, R-Square = 39% for Negative Affect and 33% for Positive Affect)

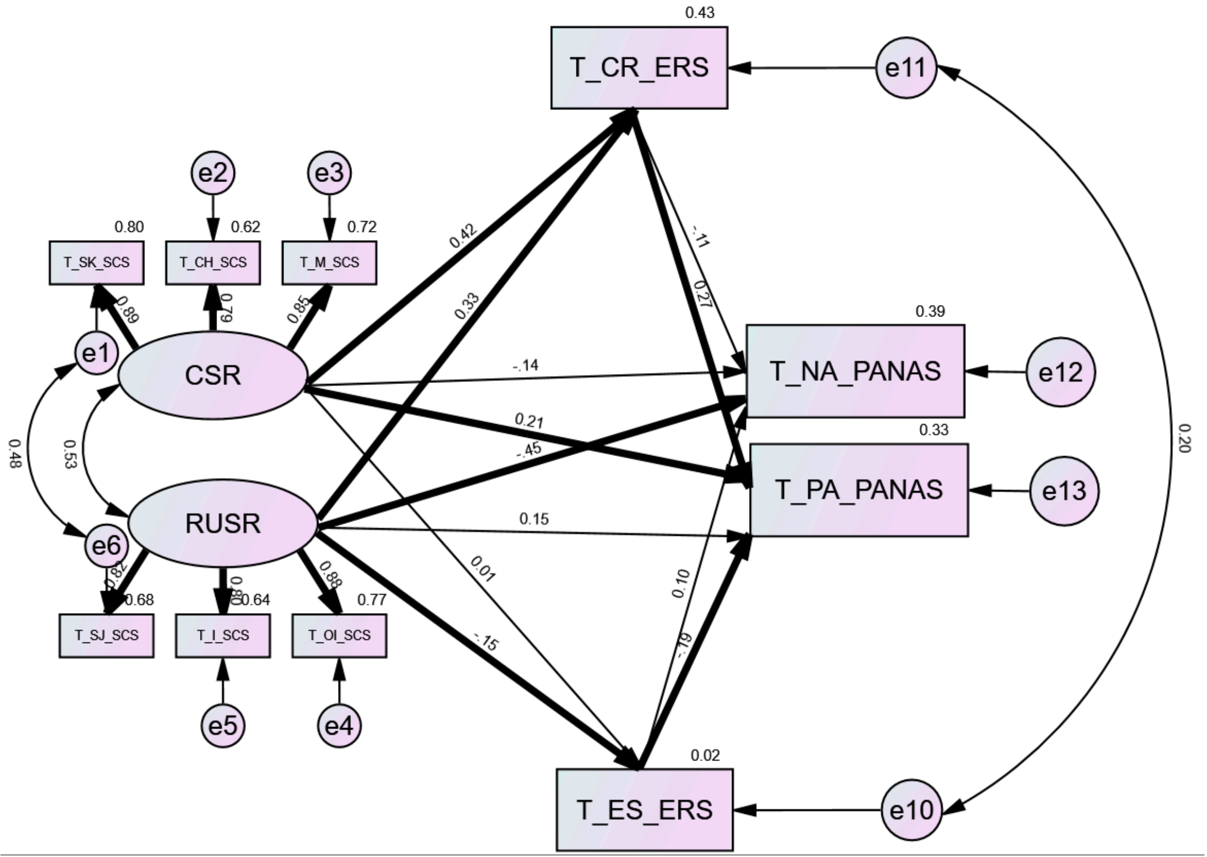

Figure S1: Structural Model with Standardised Weights and Significant Paths Bolded.

Table S1: Standardised Effect Sizes

From	To	Direct	Indirect (Total)	Total
RUSR	PAffect	.149	.117**	.266**
CSR	PAffect	.213*	.110*	.323**
RUSR	NAffect	-.448**	-.051	-.499**
CSR	Naffect	-.139	-.044	-.183*

*p<.05, ** p<.01

SWL Model (Normed Chi-Square = 3.283, GFI=.924, TLI=.897, CFI=.945, RMSEA=.115, R-Square = 34% for SWL.

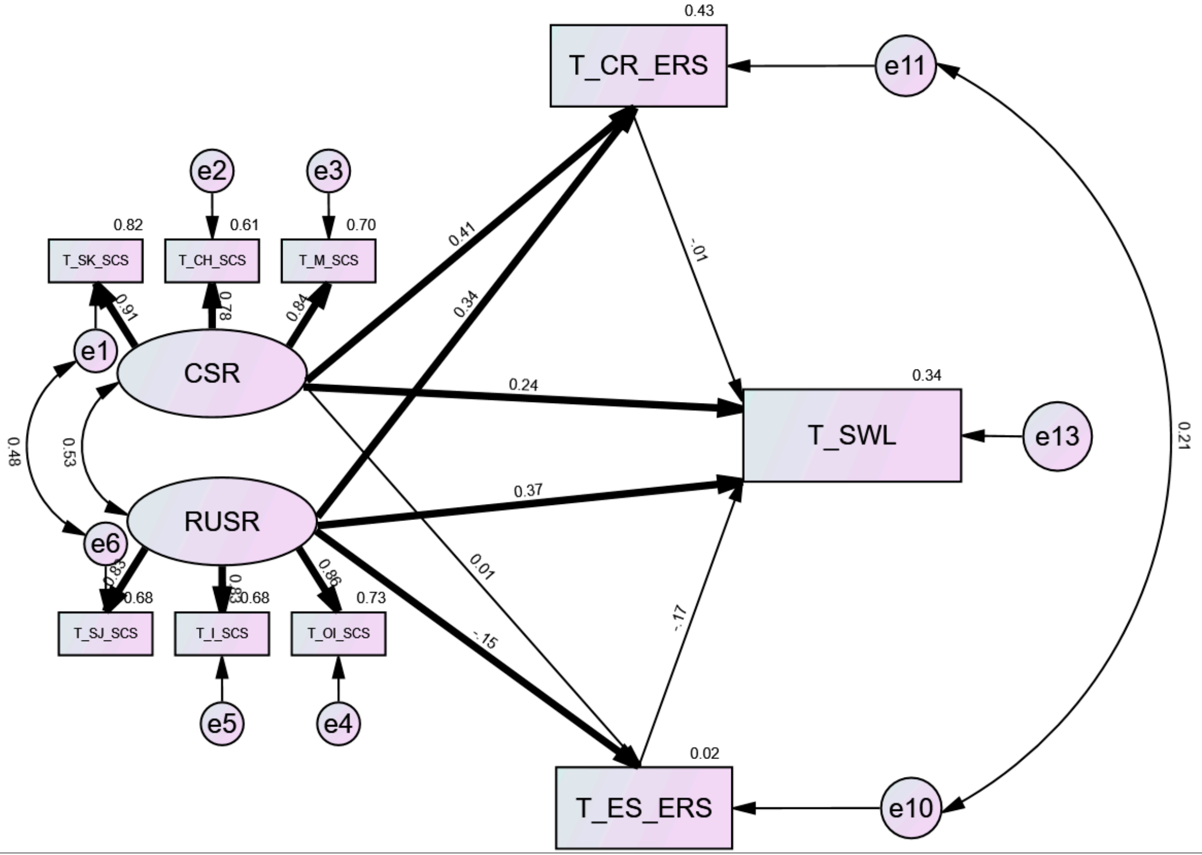

Figure S2: Structural Model with Standardised Weights and Significant Paths Bolded.

A poor fit because we don't have discriminant validity between SWL and several other constructs. See modification indices below. No indirect effects are significant.

Table S2: Standardised Effect Sizes

From	To	Direct	Indirect (Total)	Total
RUSR	SWL	.372**	.022	.394**
CSR	SWL	.242**	-.005	.237**

*p<.05, ** p<.01

Internal

Modification Indices (Group number 1 - Default model)

Covariances: (Group number 1 - Default model)

			M.I.	Par Change
e3	<-->	e10	10.281	.633
e3	<-->	e5	8.696	-.076
e3	<-->	e4	4.877	.051

Variances: (Group number 1 - Default model)

	M.I.	Par Change

Regression Weights: (Group number 1 - Default model)

			M.I.	Par Change
T_I_SCS	<---	T_M_SCS	4.664	-.126
T_M_SCS	<---	T_ES_ERS	10.815	.025

Personal Well-being - a reasonably good model fit (Normed Chi-Square = 2.194, GFI=.950, TLI=.948, CFI = .972, RMSEA=.083, R-Square for PWB = 50%)

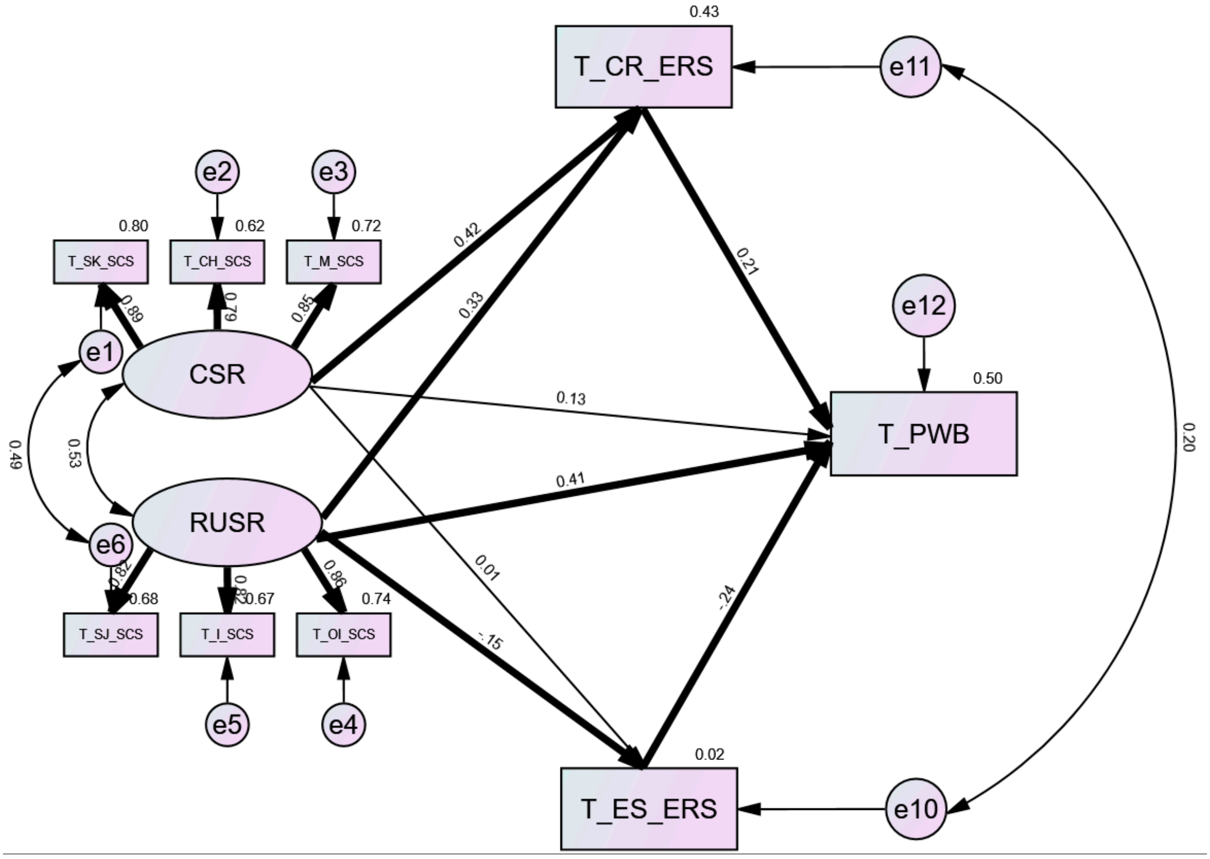

Figure S3: Structural Model with Standardised Weights and Significant Paths Bolded.

Table S3: Standardised Effect Sizes

From	To	Direct	Indirect (Total)	Total
RUSR	PWB	.411**	.108**	.519**
CSR	PWB	.133	.085	.219**

*p<.05, ** p<.01

SQRT(DASS) a reasonably good fit (Normed Chi-Square = 1.90, GFI=.955, TLI=.959, CFI=.979, RMSEA=.073, R-Square for SQRT(DASS) = 50%.

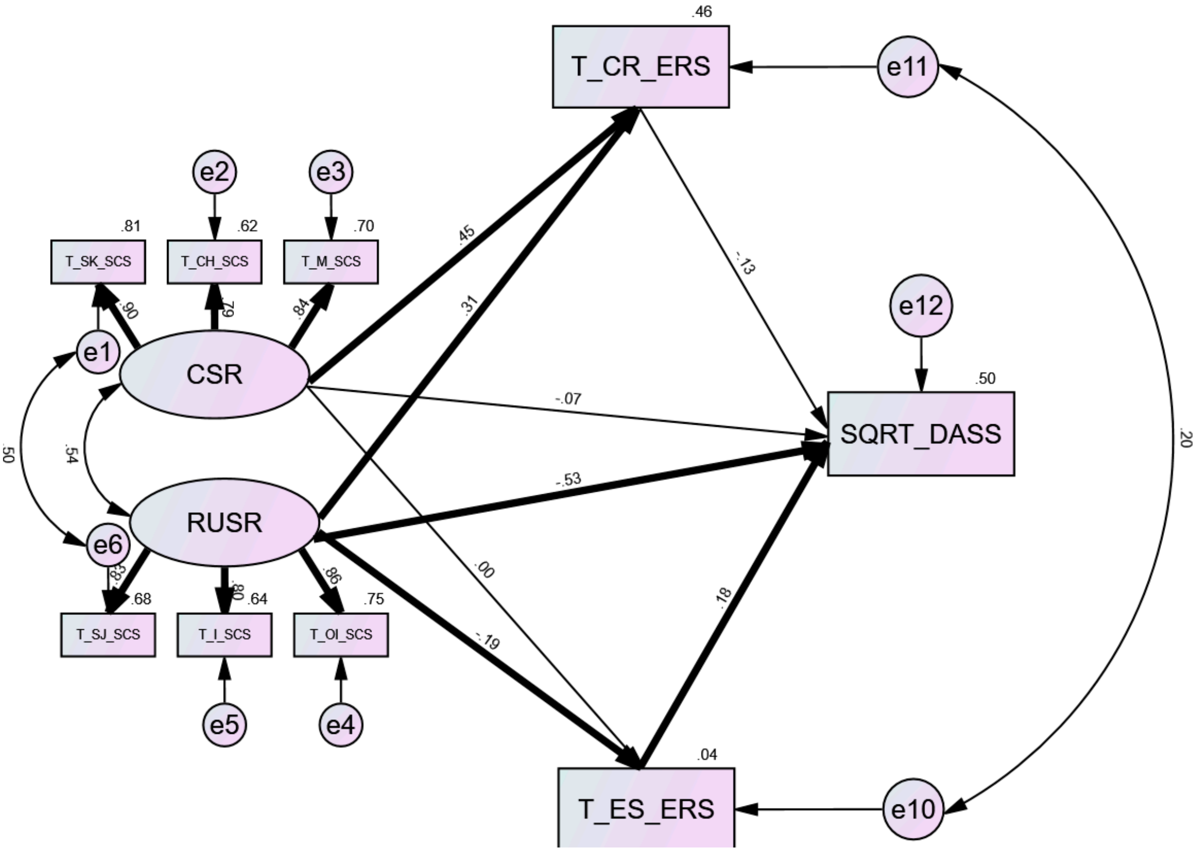

Figure S4: Structural Model with Standardised Weights and Significant Paths Bolded.

Table S4: Standardised Effect Sizes

From	To	Direct	Indirect (Total)	Total
RUSR	SQRT(DASS)	-.525**	-.074*	-.599**
CSR	SQRT(DASS)	-.056	-.056	-.112

*p<.05, ** p<.01
